# Condensation Coefficient Modulation: An Unconventional Approach to the Fabrication of Transparent and Patterned Silver Electrodes for Photovoltaics and Beyond

**DOI:** 10.1021/acsaem.4c01092

**Published:** 2024-08-20

**Authors:** Silvia Varagnolo, Ross A. Hatton

**Affiliations:** † School of Engineering and Innovation, The Open University, Walton Hall, MK7 6AA Milton Keynes, U.K.; ‡ Department of Chemistry, University of Warwick, CV4 7AL Coventry, U.K.

**Keywords:** selective metal deposition, condensation coefficient, transparent metal electrodes, thin-film photovoltaics, microcontact printing

## Abstract

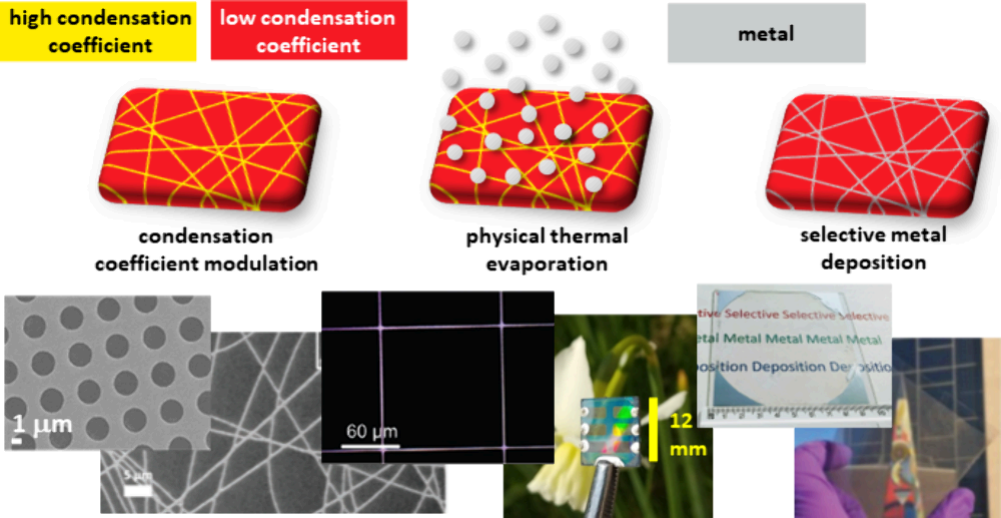

Silver is the metal of choice for the fabrication of highly transparent grid electrodes for photovoltaics because it has the highest electrical conductivity among metals together with high stability toward oxidation in air. Conventional methods for fabricating silver grid electrodes involve printing the metal grid from costly colloidal solutions of nanoparticles, selective removal of metal by etching using harmful chemicals, or electrochemical deposition of the silver, an inherently chemical intensive and slow process. This Spotlight highlights an emerging approach to the fabrication of transparent and patterned silver electrodes that can be applied to glass and flexible plastic substrates or directly on top of a device, based on spatial modulation of silver vapor condensation. This counterintuitive approach has been possible since the discovery in 2019 that thin films of perfluorinated organic compounds are highly resistant to the condensation of silver vapor, so silver condenses only where the perfluorinated layer is not. The beauty of this approach lies in its simplicity and versatility because vacuum evaporation is a well-established and widely available deposition method for silver and the shape and dimensions of metallized regions depend only on the method used to pattern the perfluorinated layer. The aim of this Spotlight is to describe this approach and summarize its electronic applications to date with particular emphasis on organic photovoltaics, a rapidly emerging class of thin-film photovoltaics that requires a flexible alternative to the conventional conducting oxide electrodes currently used to allow light into the device.

## 1 Introduction

Emerging thin-film photovoltaic (PV) devices based on organic semiconductors, organic photovoltaics (OPVs), have a photoactive layer thickness on the order of 100 nm, which is 3 orders of magnitude lower than the thickness of the silicon layer used in conventional silicon PVs.^1^ Due to this very low photoactive layer thickness and the molecular nature of organic semiconductors, OPVs can be extremely lightweight, flexible, and semitransparent.^2−4^ Hence, they are suitable for a range of applications where conventional silicon PVs cannot be installed because of their weight, rigidity, and/or optical opaqueness, such as on the surfaces of vehicles or on the windows of buildings.^5,6^ In addition to offering the combination of high transparency and low sheet resistance, the transparent electrode used in OPVs should be compatible with flexible plastic substrates not only to improve application functionality but also to be compatible with roll-to-roll high-volume production.^7^ The OPV fabrication process needs to be low-cost in terms of the processing methods and materials used, as well as to be scalable to a large area to ensure a large cost advantage and very short energy payback time over the types of emerging thin-film PVs.

To date, the transparent electrode most widely adopted in published reports on OPVs is an indium–tin oxide (ITO)-coated glass substrate fabricated by sputter deposition of ITO onto glass followed by annealing at >300 °C to achieve high transparency (∼90%) and low sheet resistance (15 Ω sq^–1^).^8,9^ Annealing at >300 °C is essential to obtain this excellent performance, making ITO incompatible with low-cost, flexible transparent plastic substrates. Furthermore, the commercially available ITO films on plastic are extremely fragile due to the intrinsically brittle ceramic nature of ITO. Many flexible alternatives to ITO glass have been proposed including electrodes based on conducting polymers,^10,11^ ultrathin metal films,^12,13^ metal nanowires,^14−16^ and metal grids.^17−19^ However, very few achieve performances comparable to that of ITO-coated glass,^20,21^ with those based on random arrays of solution-processed silver (Ag) nanowires or Ag grids offering the closest performance to that of ITO glass.^20−22^ While recent advances in the deposition of Ag nanowire electrodes onto plastic have yielded impressive performances,^22^ the synthesis of Ag nanowires is relatively costly and the nanowire films often exhibit poor contact stability at the junction between nanowires.^20,21,23,24^ While Ag grid electrodes do not suffer from the same source of electrode instability, conventional fabrication methods for Ag grids have significant disadvantages: (i) printing Ag grids from colloidal solutions of Ag nanoparticles is costly due to the relatively high cost of Ag nanoparticle synthesis;^25,26^ (ii) patterning Ag films to make grids by selective removal of Ag by chemical etching invariably uses harmful chemicals;^27^ (iii) electrochemical deposition of Ag grids is an inherently chemical-intensive and slow process.^28,29^ Recently, an innovative fabrication technique has been developed to produce Ag grids by bubble-assisted electrode assembly.^30^ This method uses almost 100% of the metallic ink and provides high-performing flexible electrodes,^30^ although the process of electrode fabrication is relatively complex and the scalability is yet to be proven.

This Spotlight article describes an unconventional and highly effective approach for the fabrication of patterned Ag electrodes based on the selective deposition of Ag vapor onto a receiving substrate by condensation coefficient modulation.^31−36^ While selective deposition of other metals has been known for some time (as discussed in section 2 of this Spotlight), it was not until 2019 that it was reported for the most electrically conductive metals Ag and copper (Cu).^31^ The key to achieving selective deposition of these low-vapor-pressure, high-melting-point metals was the use of printed films of highly fluorinated organic molecules and polymers, which were found to be highly resistant to condensation of these metals.^31−34^ Since then, this approach has been extended to include the use of vacuum-evaporated perfluorinated molecules.^35,36^ Ag vapor is produced when Ag metal is heated in a vacuum at temperatures above its melting point. When a receiving substrate (e.g., glass or plastic) at a temperature below the melting point of Ag is placed in the line-of-sight of the source of Ag vapor, metal atoms condense on the substrate. The proportion of Ag atoms incident on the substrate that remain on the surface is given by the condensation coefficient, *C*, which is the ratio of the number of adsorbed metal atoms to the total number of metal atoms arriving at the surface (i.e., when *C* = 1, 100% of incident atoms remain on the substrate).^31,37,38^ A patterned Ag film is formed by evaporating Ag onto a substrate with *C* = 1 that is patterned with a layer of perfluorinated organic compounds for which *C* can be close to zero. The Ag pattern therefore results from the spatial modulation of *C*. Because Ag is deposited only where it is needed, there is no metal removal step, which avoids metal waste and eliminates the adverse environmental impact associated with the use of chemical etchants and also leaves a pristinely clean Ag surface.^31−34^

As discussed in subsequent parts of this Spotlight, this approach has hitherto been applied to the fabrication of transparent substrate electrodes for OPV devices based on embedded Ag grid electrodes^33^ and fused Ag nanowire electrodes.^32^ It has also been applied to the fabrication of patterned Ag electrodes directly on the top of OPVs^31^ and organic-light-emitting diodes (OLEDs),^35^ as well as transparent heaters.^32^ However, there are many other applications in modern science and technology that stand to benefit from using this approach to patterning Ag, which this Spotlight will hopefully encourage.

## 2 Condensation Coefficient (*C*)

**Figure 1:**
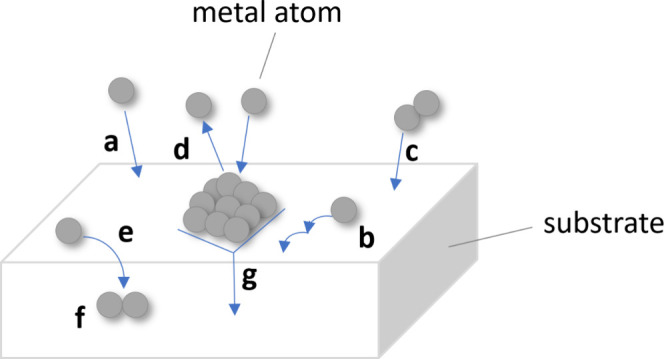
Schematic diagram of the basic processes occurring in the initial stages of metal/substrate interface formation by physical vapor deposition, where the substrate can be a polymer or can be coated with a layer of organic molecules: (a) adsorption; (b) surface diffusion; (c) nucleation and growth of critical clusters; (d) desorption; (e) diffusion into the bulk; (f) aggregation in the bulk; (g) embedding of metal clusters into the polymer. Adapted with permission from ref (38). Copyright 2012 Taylor and Francis.

At the initial stages of metal evaporation onto a substrate comprising a polymer or coated with a layer of organic molecules, metal atoms can undergo a number of different interaction processes with the surface and other incoming metal atoms (Figure 1), including adsorption at the surface, surface diffusion, bulk diffusion, nucleation, and aggregation.^37,38^ While desorption of metal atoms back into the gas phase is also possible,^37,38^ experience and conventional wisdom dictate that the fraction of metal atoms incident on the substrate that leave without condensing is typically very small when the temperature of the substrate is close to room temperature.

**Figure 2:**
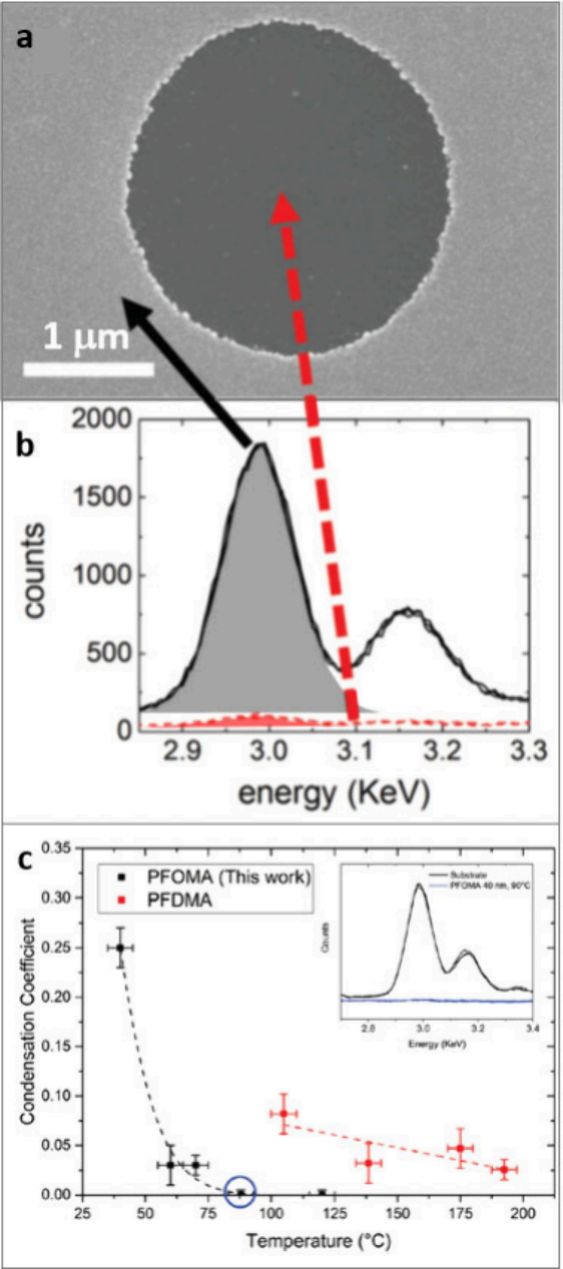
Evaluation of *C* from EDXS. (a) Scanning electron microscopy (SEM) image of a Ag layer deposited on a high-*C* substrate decorated with circular features made of a low-*C* material. (b) Corresponding EDXS spectra. The two peaks refer to X-ray transitions Lα_1_ (2.98 keV) and Lβ_1_ (3.15 keV). *C* is determined from the ratio of the Lα_1_ Ag peak areas.^31^ Reproduced from ref (31). Available under a CC-BY 3.0 license. Copyright 2019, Varagnolo et al. (c) Graph showing *C* versus substrate temperature for an Ag thickness equivalent to 50 nm deposited at 2.7 Å s^–1^ onto printed areas of PFOMA (black)^33^ and PFDMA (red).^34^ Inset: Example EDXS analysis of the data point circled in blue at 90 °C where the Ag signal was below the detectable limit.^33^ Reproduced from ref (33). Available under a CC-BY 4.0 license. Copyright 2023, Bellchambers et al.

To date, *C* for metals condensing on polymer/organic surfaces has been determined using a radiotracer technique,^37,39^ X-ray photoelectron spectroscopy (XPS),^37,38,40^ and energy-dispersive X-ray spectroscopy (EDXS).^31−34^ While radiotracer and XPS measurements can be used to determine *C* at the early stages of metal nucleation, EDXS is the method of choice when the metal thickness is greater than 10 nm. Using the most intense element-specific peak in the EDXS spectrum, *C* is determined as the ratio of the peak intensity acquired from a region coated with material designed to resist Ag condensation to that acquired from an adjacent region that is known to have *C* = 1, as illustrated in Figure 2a,b.^31−34^

Selective deposition of metal vapor onto organic substrates by condensation coefficient modulation to form patterned metal films was first reported by Tsujioka et al. for the low-melting-point (*m*_p_), high-vapor-pressure metals magnesium (*m*_p_∼ 247 °C), manganese (*m*_p_ ∼ 572 °C), lead (*m*_p_ ∼ 428 °C), zinc (*m*_p_ ∼ 177 °C), and calcium (*m*_p_ ∼ 357 °C) on thick-film (≥2 μm) poly(dimethoxysiloxane) (PDMS), a cross-linked viscoelastic polymer well-known for its chemical stability.^41,42^ Selective deposition of higher-melting-point metals gallium (*m*_p_ ∼ 742 °C), indium (*m*_p_ ∼ 597 °C), tin (*m*_p_ ∼ 807 °C), and aluminum (*m*_p_ ∼ 821 °C) was also achieved but for a low metal deposition rate.^43^ However, Ag, which has a substantially higher *m*_p_ of ∼958 °C, was found to condense on PDMS, even for a very low deposition rate.^41,42^ In 2019, it was discovered by Varagnolo and Hatton et al.,^31^ that perfluorinated polymer films with thicknesses as low as ∼10 nm were highly resistant to Ag condensation such that *C* very close to zero was possible, opening the door to the fabrication of patterned Ag electrodes by condensation coefficient modulation.^32−34,35^ In the same study, it was shown that perfluorinated polymers are also resistant to Cu condensation, which is the second most electrically conductive metal after Ag and (like Ag) is also a high-melting-point, low-vapor-pressure metal. Ag and Cu are the dominant current-carrying elements in modern electronics and PVs and the metals of choice for a diverse range of emerging applications including flexible transparent electrodes and as platforms for biological and chemical sensors for point-of-use healthcare and environmental monitoring,^44−47^ so the ability to pattern these metals by condensation coefficient modulation represents an important technological advancement. Since then, it has been shown that films of perfluorinated compounds also resist condensation of other high-melting-point, low-vapor-pressure metals, including chromium and nickel, although the maximum metal thickness that can be achieved is limited to much less than that possible with Ag, which is limiting for practical application.^36^

While past research efforts aimed at finding materials to maximize *C* for metals (including Ag) on polymer substrates are relatively common,^37−39,48−50^ reports relating to the identification of low-*C* materials and the determination of the key ingredients to suppress metal condensation are much rarer^31−34^ (Table 1). The body of evidence to date shows that for an organic/polymer substrate to be highly resistant to metal condensation it must not have chemical moieties capable of chemical reaction with the incident metal atom, and other attractive interactions (e.g., dispersive force interactions) must be small compared to the kinetic energy of the incident metal atom. By screening different materials (Table 1) Varagnolo and Hatton et al.^31^ showed that perfluorinated moieties are essential to resist Ag condensation, which is rationalized by the expectation that perfluorinated moieties interact only very weakly with the incident Ag atoms due to (i) the very high strength of the C–F bond, which makes it resistant to chemical reaction with the incident Ag atoms, and (ii) the electronegativity of F atoms, which results in the C–F bonds having exceptionally low polarizability and thus only very weak dispersive interactions with the incident Ag atoms. That study also showed that the perfluorinated layer must have a minimum critical thickness to resist Ag condensation, which depends on the fluorinated material in question, but is on the order of 10 nm.^31^ For instance, monolayers of the perfluorinated molecules perfluorooctyldimethylchlorosilane and tridecafluorooctanethiol (Table 1) do not resist Ag condensation, and films of perfluorooctyltrichlorosilane were found to resist Ag condensation only when thicker than 8.4 ± 1.6 nm.^31^ A subsequent study using evaporated films of the commercial perfluoropolyether KY-1901 (Shin-Etsu Chemical Co., Japan) found the critical thickness to be 4.6 nm.^36^ Another factor that is believed to play a role in assisting the desorption of Ag atoms from perfluorinated films is the motion of the perfluorinated parts of the polymer (or molecule) during Ag deposition: Bellchambers and Hatton et al.^33^ and Varagnolo and Hatton et al.^34^ have shown that *C* decreases sharply with increasing substrate temperature when Ag is deposited onto poly(methyl methacrylate)s with perfluorinated side chains attached via flexible −(CH_2_)_2_– linkages (Figure 2c). Movement of these side chains is expected to be strongly correlated with increasing temperature and is possible at low temperatures because of the large free volume that results from the repulsive Coulombic interactions between fluorine (F) atoms and steric hindrance effects.^51,52^ Furthermore, in general, a lower *C* for metals is exhibited by softer more fluid perfluorinated polymer surfaces.^33,42,53^ However, it is notable that the poly(vinylidene fluoride) derivative PVDF-HFP (Table 1) has also been reported to be capable of resisting Ag condensation.^31^ Given that PVDF-HFP is free of flexible perfluorinated side groups and is semicrystalline at room temperature,^54^ this finding indicates that motion of the fluorinated parts of the organic surface may yet prove to be a nonessential requirement to resisting Ag condensation.

**Table 1:**
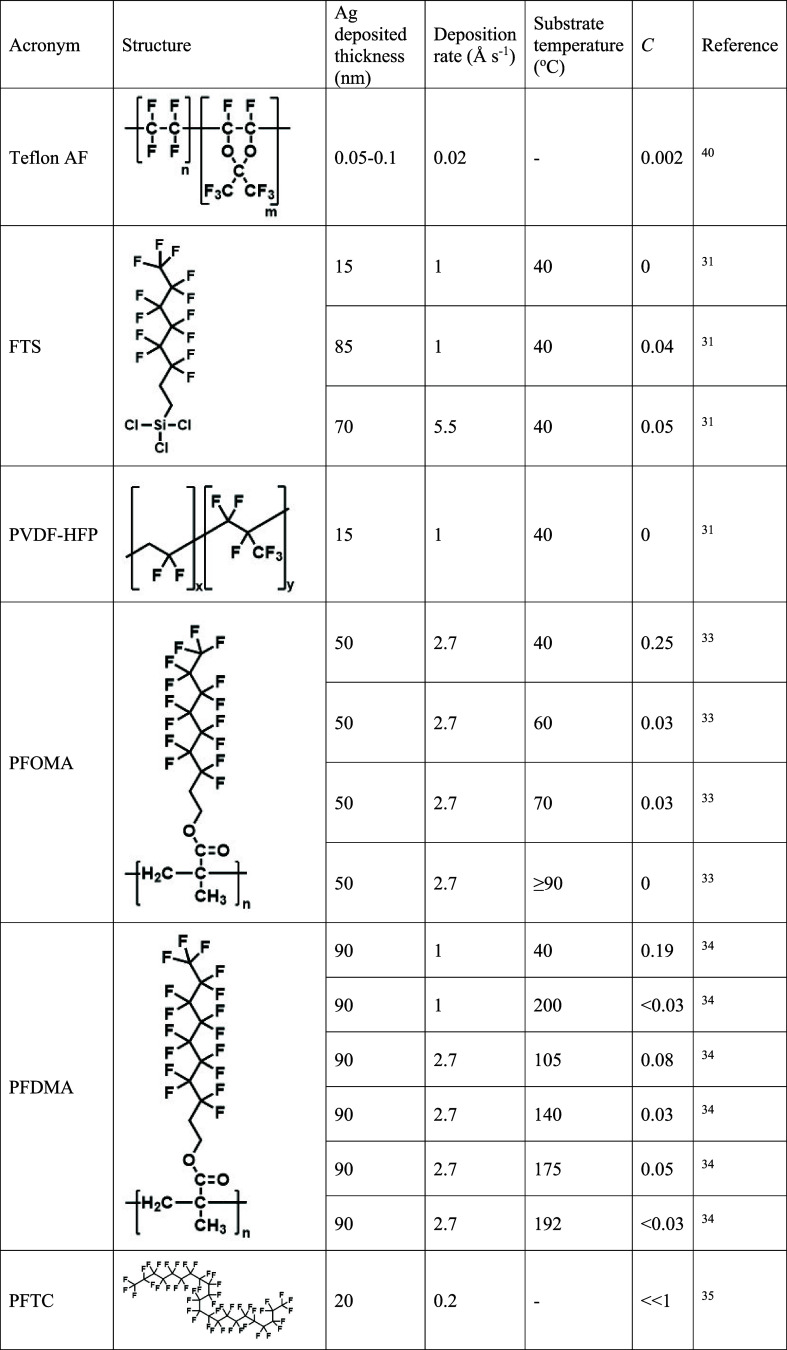
Compounds That Achieved *C* Close to 0 for Ag

## 3 Fabricating Transparent Ag Electrodes by Condensation Coefficient Modulation

A transparent Ag electrode is fabricated by condensation coefficient modulation when the receiving glass or transparent plastic substrate is prepatterned so there are isolated areas of a transparent material having *C* ∼ 0 (e.g., areas with a printed perfluorinated polymer layer) and an interconnected network of areas with *C* ∼ 1 (e.g., exposed glass or plastic). Upon exposure of the whole surface to Ag vapor, metal condenses only in the regions where *C* ∼ 1, forming a continuous electrically conductive Ag network. In practice, while *C* can be very close to zero for Ag vapor interacting with perfluorinated films, when the Ag thickness in regions where *C* ∼ 1 is increased to ≥100 nm, isolated Ag nanoparticles can begin to form on the perfluorinated layer, the density and size of which increases with increasing Ag deposition time and rate,^31−34^ the reason for which remains the subject of ongoing research. However, due to the large absorption cross section of Ag nanoparticles that results from localized surface plasmon excitation,^27−29^ Ag nanoparticles that form when an equivalent Ag thickness as little as a fraction of a nanometer condenses on the perfluorinated layer (e.g., *C* = 0.005) can still result in significant parasitic absorption. Fortunately, due to their very small size, these nanoparticles can be easily removed with a brief solvent rinse or a UV/O_3_ treatment followed by rinsing with acetic acid, which oxidizes and dissolves the Ag nanoparticles, respectively.^32−^ Adventitiously, UV/O_3_ treatment also oxidizes the surface of the perfluorinated layer, increasing its surface energy so that it can be wetted with the conducting polymer poly(3,4-ethylenedioxythiophene):poly(styrenesulfonate) (PEDOT:PSS; PH1000), which is sufficiently conductive to span the gaps between grid lines even when very thin (<30 nm).^17^ The different micro- and nanofabrication techniques applied to date to form transparent and patterned Ag electrodes by condensation coefficient modulation are described in the following subsections.

### 3.1 Microcontact Printing (μCP)

**Figure 3:**
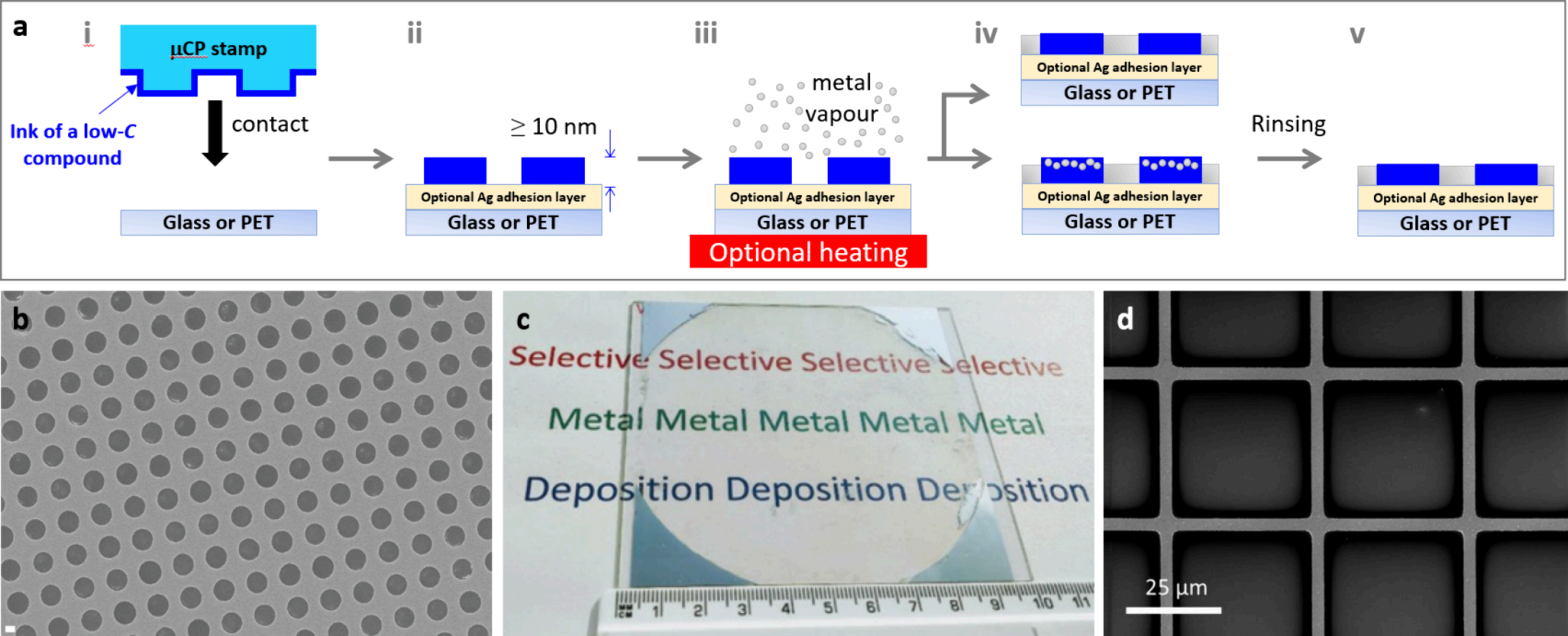
Ag patterns by condensation coefficient modulation through μCP. (a) Schematic of the selective metal deposition process based on a microcontact-printed low-*C* layer: (i) a PDMS stamp having micron-sized pillars inked with a solution of an organofluorine compound and brought into contact with the substrate optionally coated with a high *C* adhesive layer for the metal (molybdenum oxide or polyethylenimine); (ii) a printed pattern of an organofluorine metal-repellent layer with a thickness of ≥10 nm; (iii) metal deposition over the whole substrate by vacuum thermal evaporation; (iv) selective condensation of Ag mainly where the organofluorine layer is not [Ag might condense only on the uncoated substrate (top) or Ag nanoparticles might deposit on the organofluorine layer (bottom)]; (v) optional rinsing process to remove the Ag nanoparticles.^31,33,34^ Adapted from ref (31). Available under a CC-BY 3.0 license. Copyright 2019, Varagnolo et al. (b) SEM image of an 85-nm-thick Ag film on MoO_3–*x*_/glass with 2.5-μm-diameter circular apertures where FTS is printed. The scale bar corresponds to 1 μm.^31^ Reproduced from ref (31). Available under a CC-BY 3.0 license. Copyright 2019, Varagnolo et al. (c) Picture of a 10-cm-diameter hole in a 50-nm-thick Ag film fabricated by printing an FTS layer using a PDMS stamp on a MoO_3–*x*_ (15 nm)/glass substrate.^31^ Reproduced from ref (31). Available under a CC-BY 3.0 license. Copyright 2019, Varagnolo et al. (d) SEM images showing a 40 μm pitch Ag square grid as deposited.^33^ Reproduced from ref (33). Available under a CC-BY 4.0 license. Copyright 2023, Bellchambers et al.

A particularly successful approach to transparent Ag electrode fabrication by condensation coefficient modulation is to use μCP to deposit a patterned perfluorinated layer onto a substrate for which *C* is ∼1, such as glass or poly(ethylene terephthalate) (PET).^31,33,34^ The receiving substrate can also be modified over its whole surface with a Ag nucleation layer such as molybdenum oxide or polyethylenimine prior to μCP.^31,33,34^ μCP uses a viscoelastic stamp made of PDMS with elevated features loaded with the material to be printed. When the loaded stamp is bought into contact with the receiving substrate an intimate conformal contact is made between them, ensuring efficient transfer of the material on the stamp to the substrate.^55,56^ Provided the energy of adhesion between the printed film and receiving substrate is greater than that between the PDMS and the printed film, transfer will occur, which for perfluorinated compounds can be essentially instanteous.^33,57^ μCP is attractive because it can be scaled to large areas and is easily implementable on a laboratory scale.^55,56,58^ Transparent Ag grid electrodes can be fabricated by μCP perfluorinated polymers with a thickness of ≥10 nm onto a receiving substrate with high *C* for Ag [Figure 3a(i,ii)]. The printed surface is then exposed to Ag vapor [Figure 3a(iii)], forming an Ag grid that is partially or fully embedded into the perfluorinated layer depending on the thickness of the latter [Figure 3a(iv)]. If required, a simple solvent rinse enables the removal of any Ag nanoparticles on the perfluorinated layer [Figure 3a(v)]. Notably, the highly fluorinated molecules and polymers used to achieve selective Ag condensation are soluble in perfluorinated or highly fluorinated solvents (e.g., HFE-7500), which are typically orthogonal solvents for the oxide charge extraction layers (e.g., zinc oxide) and organic semiconductors used in organic electronics. Consequently, the optional rinsing step can be performed even when the deposition of Ag by condensation coefficient modulation is performed on top of an organic optoelectronic device, as described in section 4.3.

To date, organofluorine compounds used as μCP inks include FTS, PFDMA, and PFOMA (Table 1).^31,33,34^ The small molecule FTS is extremely effective in suppressing Ag condensation^31^ but exhibits two drawbacks: (i) the high sensitivity of the chlorosilane moieties on FTS toward reaction with water requires a high degree of control over the water levels in both the solvent used to prepare the FTS solution and ambient air to ensure that the critical film thickness can be achieved over a large area; (ii) the number of times the PDMS stamp can be reused is limited by the propensity of FTS to polymerize particularly when the printed feature sizes are micron-sized.^34^ The polymer PFDMA provides much better control on the thickness of the printed features and allows for multiple uses of the same PDMS stamps after appropriate rinsing, although it does not resist Ag condensation as effectively as FTS.^33,34^ Bellchambers and Hatton et al.^33^ have recently shown that reducing the length of the perfluorinated chains so that it is the same as that on FTS dramatically reduces *C* for Ag, such that *C* ∼ 0 can be achieved when the substrate is heated to ≥90 °C, thereby avoiding the need for a solvent rinsing step.^33^ Crucially, 90 °C is well below the melting or deformation temperature of PET.

This technique has been used to produce Ag patterns of different shapes and dimensions including Ag films with a periodic array of 6 million 2-μm-diameter holes per square cm (Figure 3b),^31^ a single circular hole of 10 cm diameter (Figure 3c),^31^ and squared grids with a line width a factor of 10 lower than can be achieved using conventional printing techniques (i.e., screen, inkjet, and flexographic printing) using colloidal Ag inks (Figure 3d).^33,34^

### 3.2 Electrospinning

**Figure 4:**
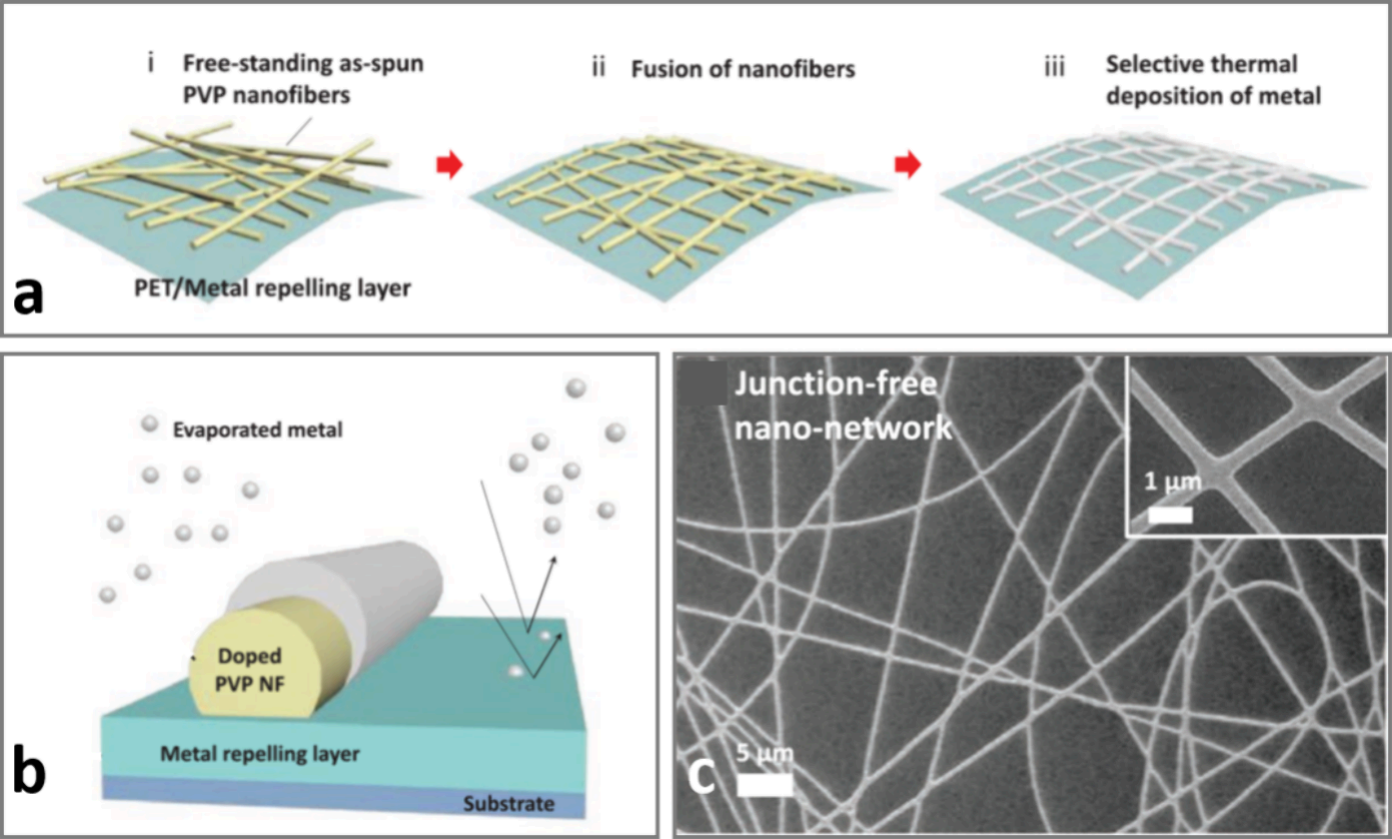
Ag NN by condensation coefficient modulation through electrospinning. (a) Illustration of the stages of metal NN fabrication: free-standing electrospun NFs (i) are fused (ii), and metal is selectively deposited onto the fused NF network (iii) by condensation coefficient modulation. (b) Schematic of methoxysilane-doped PVP NFs and the underlying Ag repelling layer composed of a blend of FTS and PFDMA. (c) SEM image of the fused Ag NN electrode, with the inset showing a magnified image from the same network. The average diameter of Ag nanowires is ≈430 nm.^32^ Adapted from ref (32). Available under a CC-BY 4.0 license. Copyright 2020, Lee et al.

High-performance transparent electrodes can also be produced by electrospinning high-*C* nanofibers (NFs) onto a substrate coated with a perfluorinated layer, followed by fusing the nanowires and Ag evaporation, forming junction-free random Ag nanowire network electrodes:^32^Figure 4. Our group (Lee et al.)^32^ first reported this approach for the fabrication of transparent Ag electrodes on flexible PET substrates using a PFDMA:FTS blend as the low-*C* layer and poly(vinylpyrrolidone) (PVP) nanowires doped with (3-mercaptopropyl)trimethoxysilane and (3-aminopropyl)trimethoxysilane as the high-*C* material.^59^ The latter two small molecules were added to PVP to serve as cross-linking agents and nucleation sites for incident Ag atoms because both thiols and primary amines have a high affinity for Ag.^50^ After ∼100 nm Ag evaporation, Ag nanoparticles were found to form in the organofluorine layer, conferring a brownish tinge to the sample, but were easily removed by rinsing in a fluorinated solvent (HFE-7500).^32^

## 4 Applications to OPVs, OLEDs, and Transparent Flexible Heaters

This section describes the device applications of Ag patterning by condensation coefficient modulation reported to date.

### 4.1 Embedded Grid Electrodes

**Figure 5:**
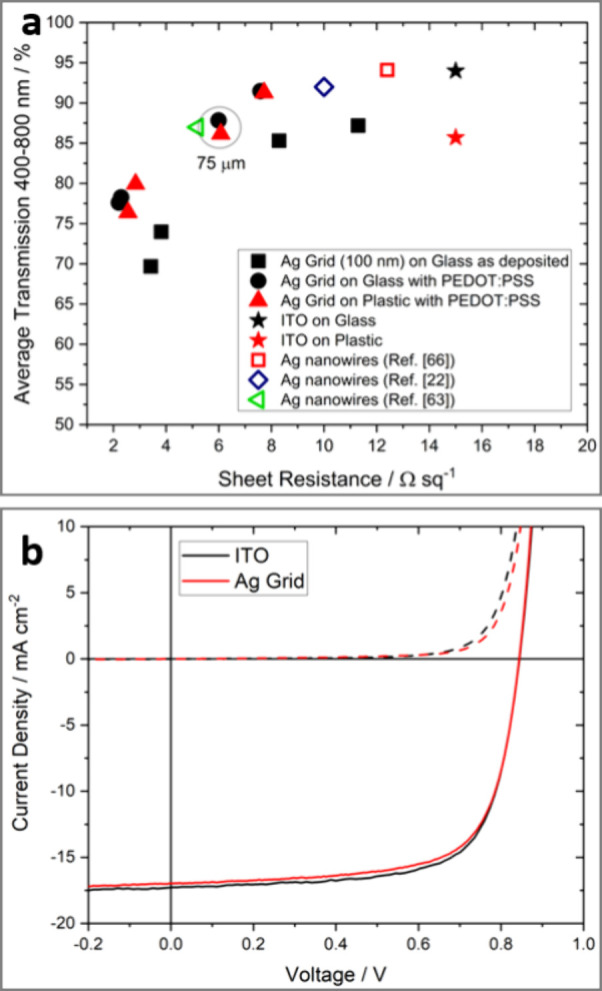
Square grid electrodes for OPVs by condensation coefficient modulation. (a) Optoelectronic performance of a range of metal grid patterns with a line width of 3 ± 1 μm, a metal thickness of 100 nm, and pitches of 40, 50, 75, and 150 μm. (b) Champion ITO and Ag grid devices for the device structure transparent electrode|ZnO|PBDB-T/ITIC|MoO_3–*x*_|Al, tested under 1-sun-simulated solar illumination (solid lines) and in the dark (dashed lines). Summary of all device data given in Table 2. The grid transparent electrode has a pitch of 75 μm, a line width of 3 ± 1 μm, and a thin PEDOT:PSS interlayer of 25 nm.^33^ Adapted from ref (33). Available under a CC-BY 4.0 license. Copyright 2023, Bellchambers et al.

Ag grid electrodes fabricated using the method described in section 3.1 were proven to exceed the performance of commercial ITO-coated glass and plastic and exhibit performances comparable to those of examples of the best-performing alternative approaches to the fabrication of Ag nanowire/nanonetwork (NN) electrodes suitable for use in organic optoelectronics: Figure 5a.^33^ Specifically, Ag grid patterns with a line width of 3 ± 1 μm, a metal thickness of 100 nm, and pitches of 40, 50, 75, and 150 μm were produced by a μCP pattern of PFOMA square features 40 nm thick with a substrate temperature of 120 °C during Ag thermal evaporation, which ensured *C* ∼ 0. The gap between grid lines was spanned with PEDOT:PSS, formulation PH1000. Advantageously, the PEDOT:PSS deposition step induces a substantial reduction in sheet resistance (e.g., from 8.3 to 6.0 Ω sq^–1^ for a 75 μm pitch grid) ascribed to an improvement in the crystallinity of the Ag grid lines upon heating at 120 °C because the PEDOT:PSS film alone has a sheet resistance of >1000 Ω sq^–1^.^33^ Importantly, there is no significant difference in the performance between Ag grid and PEDOT:PSS electrodes fabricated on glass and flexible PET substrates (Figure 5a). Furthermore, these electrodes were demonstrated to be extremely robust against repeated bending: grids on PET exhibited an increase of sheet resistance of only 5% after bending for 100000 times through a radius of curvature of 6 mm, while the sheet resistance of commercial ITO-coated flexible plastic was found to increase by a factor of 30 times after only 100 bend cycles.^33^ Additional scotch tape adhesion testing,^60^ applied three times to determine if delamination of the grid from the substrate had occurred as a result of repeated bending, induced an increase of the sheet resistance by less than 1%, offering compelling evidence that the grid lines remain strongly bound to the plastic substrate.^33^

**Figure 6:**
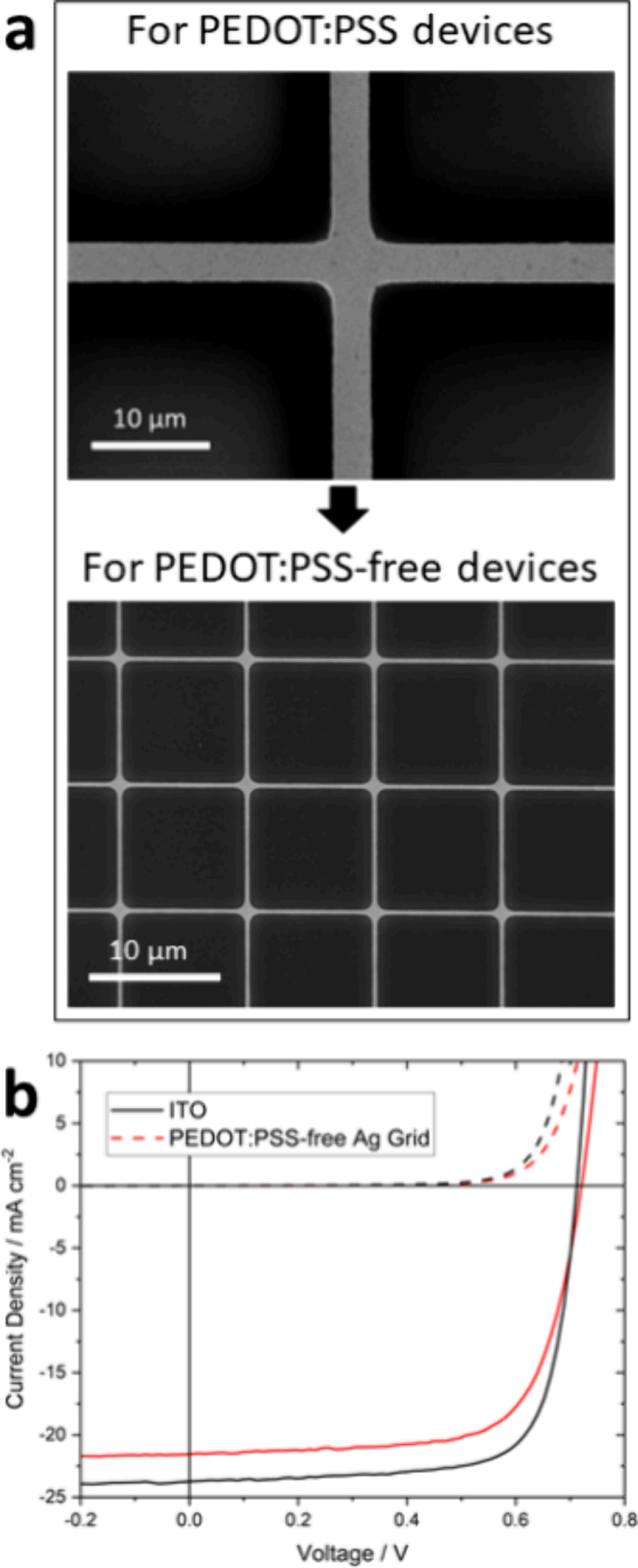
PEDOT:PSS-free devices. (a) SEM images (both scale bars 10 μm) highlighting the reduction in pitch and line width to enable PEDOT:PSS-free devices. (b) Champion ITO and PEDOT:PSS-free Ag grid devices for the device structure grid/ITO|ZnO|PM6:Y6|MoO_3_|Al. A summary of all device data is given in Table 3. Please note that these devices are based on the PM6:Y6 bulk heterojunction (a more modern derivative of PCE-12:ITIC) and so are not directly comparable to the data in Figure 5b.^33^ Adapted from ref (33). Available under a CC-BY 4.0 license. Copyright 2023, Bellchambers et al.

The Ag grid electrodes with a pitch of 75 μm (circled in Figure 5a) exhibited an average transparency of 88% for a sheet resistance of 6 Ω sq^–1^, and their utility as a drop-in replacement for ITO glass in efficient model OPV devices was demonstrated.^33^ OPV devices using the PEDOT:PSS-coated Ag grid electrode exhibited a performance comparable to that using optimized commercial ITO-coated glass, which is widely considered to be the gold standard transparent electrode for achieving high power conversion efficiency in OPVs.^33^ (Figure 5b and Table 2). In that work, the utility of Ag grid electrodes was also demonstrated in PEDOT:PSS-free OPVs because the use of PEDOT:PSS is associated with OPV device instability resulting from its acidity and propensity to take up water.^61,62^ Removing PEDOT:PSS from the structure requires the distance between adjacent grid lines (i.e., the pitch) to be reduced to avoid excessive resistive losses, and so the resulting higher grid line density has to be offset, as far as possible, by a reduction in the line width. To this end, Ag grids lines of 100 nm thickness and 10 μm pitch were reduced to 600 ± 100 nm line width (Figure 6 and Table 3), resulting in an average transparency of 85% and a conductivity of 6.0 Ω sq^–1^.^33^ This electrode was implemented in an inverted device architecture using a commercial nanoparticulate aluminum-doped ZnO electron transport layer, which has a conductivity 6 orders of magnitude below that of PEDOT:PSS (PH1000). The power conversion efficiency of model OPVs using this PEDOT:PSS-free electrode was lower than that of identical devices using conventional ITO glass due to a lower current, consistent with the lower electrode transparency, and a slightly increased series resistance associated with the reduced conductivity of Al:ZnO compared to PEDOT:PSS. However, this difference may be considered acceptable if improvements in the device stability resulting from the omission of PEDOT:PSS are forthcoming.^33^ It is noted that alternative more conductive charge extraction layers could be used to avoid this parasitic resistance altogether and/or relax the requirement for the gridlines to be so close together.^63^ Furthermore, while the thickness of the fluorinated polymer can be chosen to match the thickness of the Ag grid lines so the electrode is fully embedded,^33,34^ it is also possible to remove the fluorinated polymer without compromising the OPV device shunt resistance, provided the Ag line thickness is ≤100 nm.^33^

**Table 2:** Tabulated Device Data for the Structure Transparent Electrode|ZnO|PBDB-T/ITIC|MoO_3_|Al^a^

	*J*_sc_/mA cm^–2^	*V*_oc_/V	fill factor	PCE/%
ITO	16.8 ± 0.3 (17.3)	0.84 ± 0.01 (0.84)	0.70 ± 0.01 (0.70)	9.9 ± 0.3 (10.2)
Ag grid	16.6 ± 0.2 (17.0)	0.84 ± 0.01 (0.84)	0.68 ± 0.05 (0.70)	9.5 ± 0.7 (10.0)

a Reproduced from ref (33). Available under a CC-BY 4.0 license. Copyright 2023, Bellchambers et al.

**Table 3:** Tabulated Device Data for the Structure Grid/ITO|ZnO|PM6:Y6|MoO_3_|Al^a^

electrode	*J*_sc_/mA cm^–2^	*V*_oc_/V	FF	PCE/%
ITO	22.8 ± 1.0 (23.7)	0.71 ± 0.01 (0.71)	0.72 ± 0.02 (0.74)	11.8 ± 0.7 (12.5)
PEDOT:PSS-free Ag grid	19.7 ± 0.9 (21.3)	0.73 ± 0.01 (0.73)	0.68 ± 0.02 (0.70)	9.8 ± 0.5 (10.7)

a Reproduced from ref (33). Available under a CC-BY 4.0 license. Copyright 2023, Bellchambers et al.

### 4.2 Nanowire Electrodes for OPVs and Transparent Heaters

**Figure 7:**
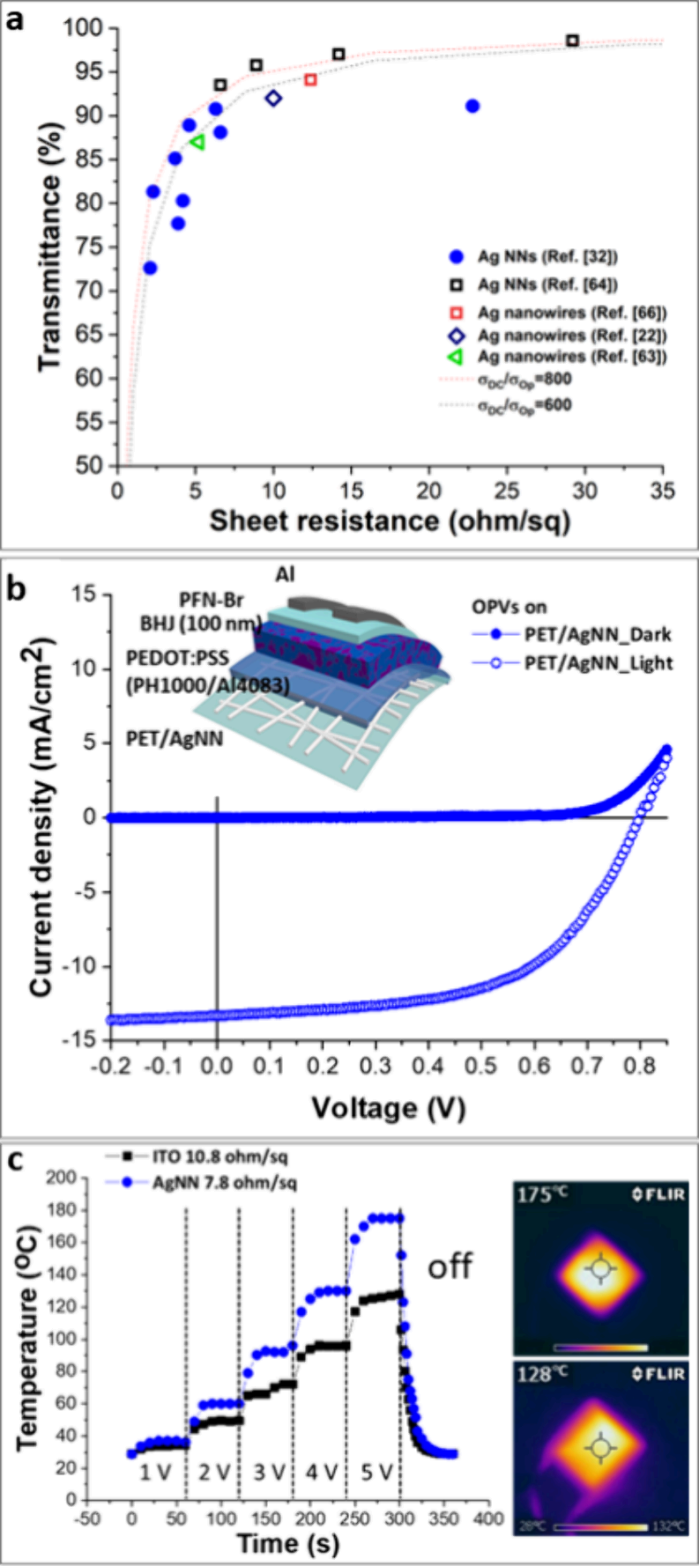
Ag NNs as electrodes for OPVs and transparent heaters: (a) average far-field transmittance over wavelength range 400–800 nm versus sheet resistance of various Ag NN electrodes fabricated in this study together with other ITO alternatives reported in the literature; (b) current–voltage characteristic of a model OPV device tested in the dark and under 1-sun-simulated solar illumination using the Ag NN on PET as a substrate electrode, together with a schematic of the device architecture; (c) performance of flexible PET/Ag NN and PEN/ITO transparent heaters at 1–5 V bias. Also shown are thermal camera images of PET/Ag NN (top right) and PEN/ITO (bottom right) at a 5 V applied potential.^32^ Adapted from ref (32). Available under a CC-BY 4.0 license. Copyright 2020, Lee et al.

Ag-fused nanowire NNs produced through the method described in section 3.2 (Figure 4) were demonstrated to be suitable as transparent electrodes for OPVs by Lee and Hatton et al.^32^ Ag nanowires with a diameter ∼430 nm and a metal thickness of ∼100 nm were found to form a continuous NN with a direct-current conductivity/optical conductivity (σ_DC_/σ_Op_) between 600 and 800, which fulfills the industrial requirements of 85% transmittance at 10–15 Ω sq^–1^ sheet resistance for PV applications and displays (Figure 7a).^32,64−66^ For a total transparency of 90.8%, the electrode sheet resistance is 6.3 Ω sq^–1^, a performance that is comparable to the best reported performance for metal nanowire electrodes fabricated using conventional top-down etching methods^64,67,68^ (Figure 7a).^32^ Such electrodes have also been demonstrated to be extremely robust toward heating up to 200 °C, bending and stretching,^32^ opening the door to their use as transparent flexible heaters.^32^ As shown in Figure 7c, the Ag nanowire heats up at a lower applied voltage than ITO and well below the 12 V used for window heaters used in automobiles.^69,70^ Furthermore, the temperature of the nanowire network electrode stabilizes more quickly than that of ITO and exhibits uniformity even upon bending.

### 4.3 Patterned Ag Electrodes Fabricated on Top of OPVs and OLEDs

**Figure 8:**
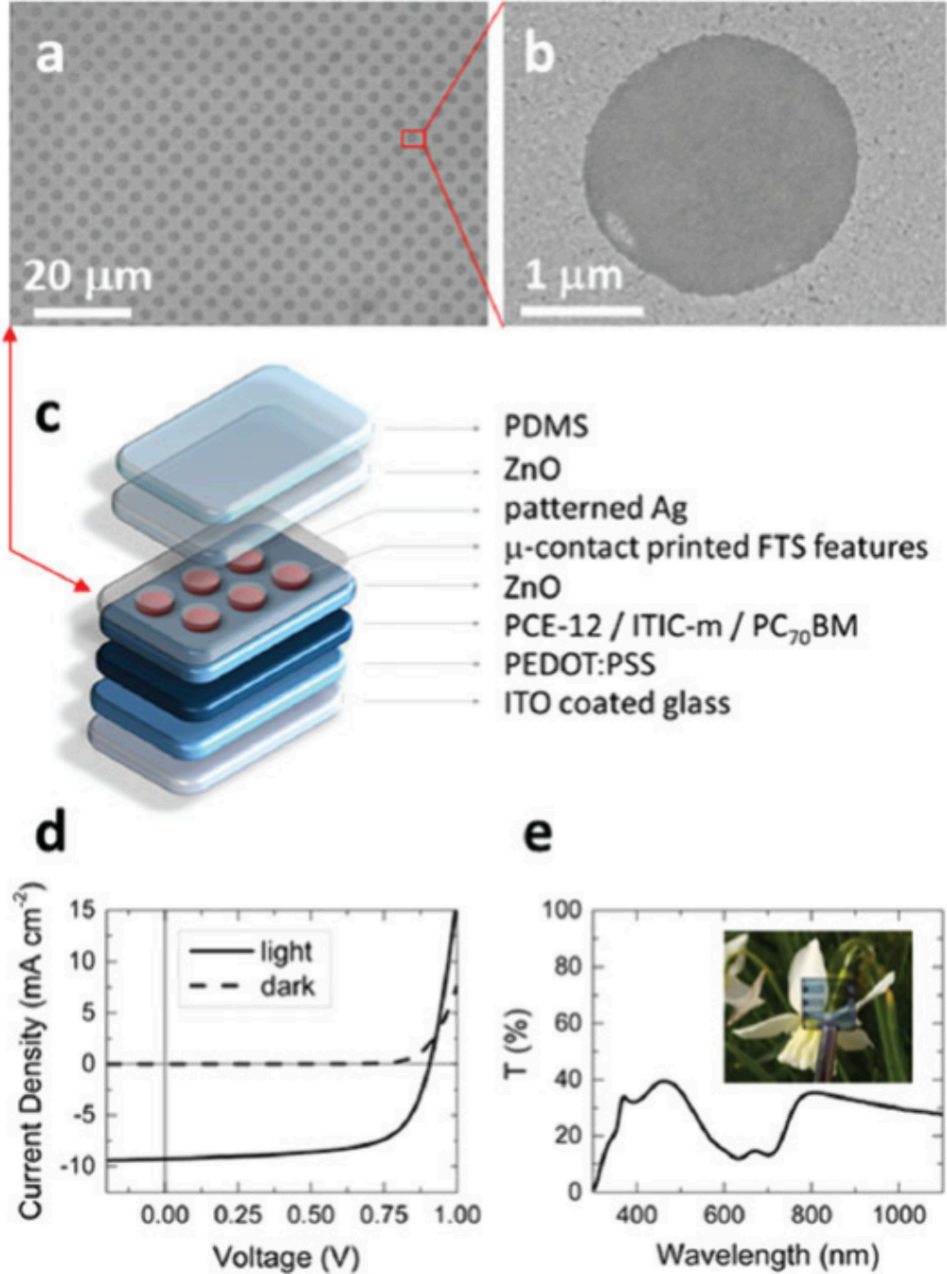
Semitransparent organic PV devices. (a and b) SEM images of the patterned Ag electrode after coating with a ZnO layer. (c) Schematic of the device architecture: glass|ITO|PEDOT:PSS|PCE-12:ITIC-m:PC_70_BM|ZnO|microcontact-printed FTS|Ag (17 nm)|ZnO|PDMS. (d) Representative current density–voltage characteristics for devices with the structure shown in part c. (e) Total transmittance (referenced to air) of the semitransparent devices with the structure shown in part c. Inset: Photograph of one device.^31^ Reproduced from ref (31). Available under a CC-BY 3.0 license. Copyright 2019, Varagnolo et al.

Condensation coefficient modulation of Ag vapor has been used to fabricate Ag electrodes directly on top of OPVs^31^ and OLEDs.^35^ Varagnolo and Hatton et al.^31^ reported the fabrication of a semitransparent electrode comprising a 17-nm-thick Ag electrode patterned with 6 million 2-μm-diameter apertures cm^–2^ directly on top of a solution-processed OPV (Figure 8), which, to our knowledge, cannot be achieved by any other scalable means directly on an organic electronic device. Such a high density of tiny holes was achieved using μCP and perfluorinated molecule FTS prior to Ag evaporation. In that case, the substrate electrode was ITO glass and the light-harvesting layer was thin enough not to absorb all of the incident light so the device was semitransparent.

**Figure 9:**
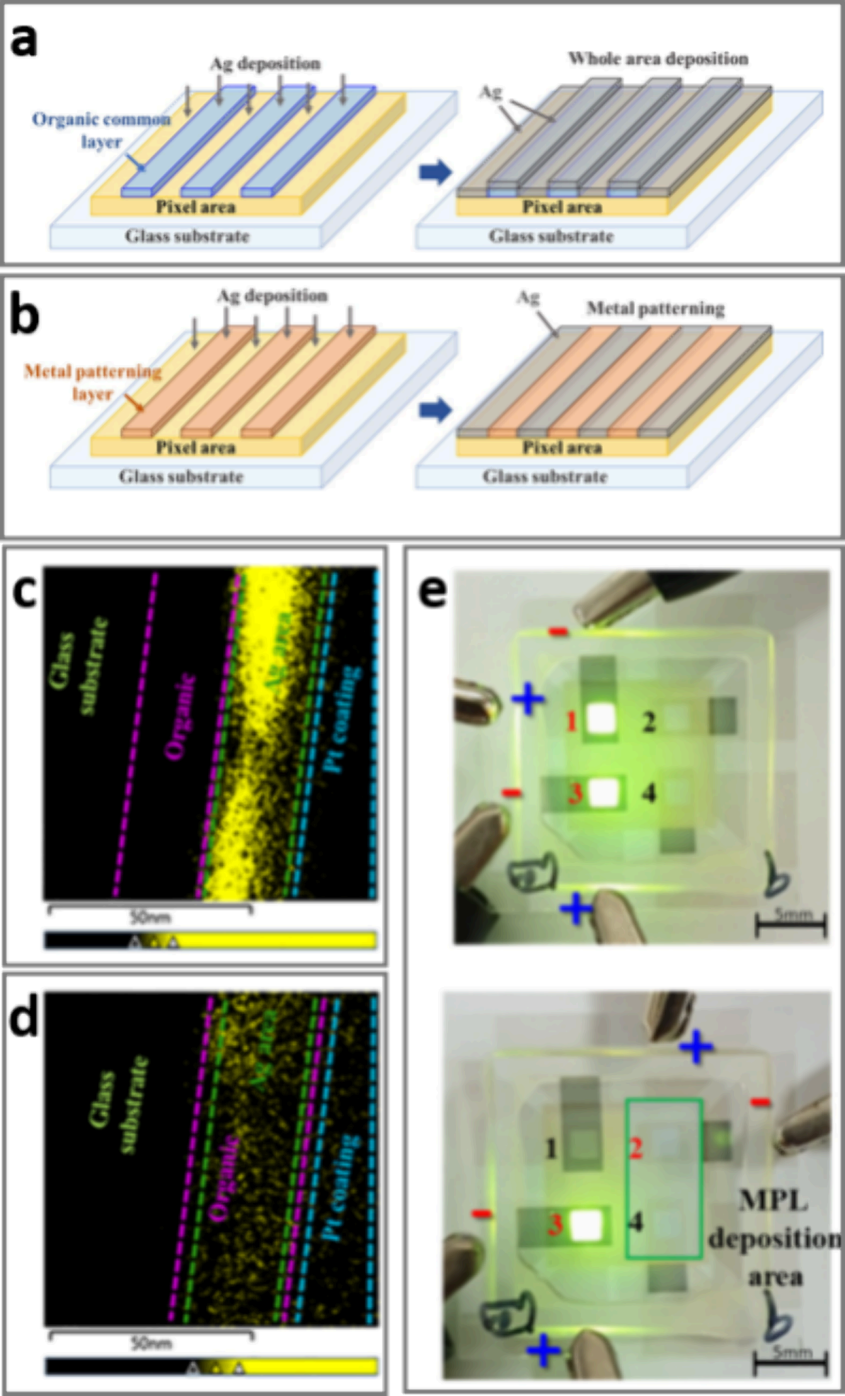
OLEDs. (a) Ag evaporation on an organic common layer resulting in Ag deposition on the whole area; (b) Ag evaporation on a metal patterning layer resulting in a metal pattern to produce the OLED cathode; (c) EDXS cross-sectional image of Ag deposited on an organic compound; (d) EDXS cross-sectional image of Ag deposited on a PFTC layer; (e) photographs of the OLED device. Reproduced with permission from ref (35). Copyright 2022 Elsevier.

For the fabrication of OLEDs, including semitransparent OLEDs, Ag is the metal of choice for the electron-injecting electrode because of its lowest light absorption and highest conductivity among metals.^35^ For application in high-performance red–green–blue OLED pixels used for information displays, Ag is typically coevaporated with the low-work-function metal magnesium in a ratio of 10:1 (Ag/Mg) to reduce the barrier to electron injection. By screening a series of different vacuum-depositable small molecules commonly used for OLED displays, Kim et al.^35^ have recently shown that, unlike the nonfluorinated molecules tested, the perfluorinated molecule poly(fluorotetracosane) (PFTC) was very effective at hindering condensation of both Ag and Mg, enabling the precise cathode patterning essential for information displays (Figure 9). Given the current and projected importance of OLEDs for display and lighting applications, this application is particularly timely.

## 5 Conclusions and Future Perspectives

This Spotlight article has described an unconventional emerging approach for the fabrication of patterned Ag electrodes for OPVs, OLEDs, and heaters, based on modulating the condensation of Ag vapor using patterned perfluorinated polymers and small molecules. The beauty of this approach lies in its versatility and simplicity because vacuum evaporation of metals is proven as a low-cost method for making thin metal films by the packaging industry, and the shape and dimensions of the features deposited are limited only by the method used to deposit the patterned organofluorine layer. This novel approach (i) can be applied to both insulating and conducting substrates, (ii) uses tiny amounts of organic compounds (the critical thickness has been shown to be as low as ∼10 nm and is 2 orders of magnitude thinner than the photoresist layers typically used in conventional photolithography), (iii) avoids the use of harmful metal etchants, and (iv) can be applied to the top surface of semiconductor devices.

Due to the versatility of this approach to Ag patterning, it is envisaged that it will prove applicable, well beyond organic optoelectronics, to displays, light-emitting diodes, PVs, wearable electronics, and sensors based on inorganic materials. Furthermore, as demonstrated by Varagnolo et al., there is also scope to substitute Ag with Cu, which offers a conductivity comparable to Ag at ∼1% of the cost. Another interesting future direction for the development of this approach might be its extension to patterning gold, which has not yet been reported. Gold is particularly attractive for use in plasmonic sensors, due to its chemical inertness, which could be applicable to many branches of chemical, biological, and biomedical applications.
